# Immunological and molecular epidemiological characteristics of acute and fulminant viral hepatitis A

**DOI:** 10.1186/1743-422X-8-254

**Published:** 2011-05-23

**Authors:** Zahid Hussain, Syed A Husain, Fahad N Almajhdi, Premashis Kar

**Affiliations:** 1 PCR Hepatitis Laboratory, Department of Medicine, Maulana Azad Medical College, New Delhi, 110002, India; 2 Center of Excellence in Biotechnology Research, King Saud University, P.O. Box 2460, Riyadh, 11451, Saudi Arabia; 3 Human Genetics Laboratory, Department of Biosciences, Jamia Millia Islamia, New Delhi, 110025, India; 4 Department of Botany and Microbiology, College of Science, King Saud University, P.O. Box 2455, Riyadh 11451, Saudi Arabia

**Keywords:** acute hepatitis A, fulminant hepatitis A, genotype, viral load, RT-PCR

## Abstract

**Background:**

Hepatitis A virus is an infection of liver; it is hyperendemic in vast areas of the world including India. In most cases it causes an acute self limited illness but rarely fulminant. There is growing concern about change in pattern from asymptomatic childhood infection to an increased incidence of symptomatic disease in the adult population.

**Objective:**

In-depth analysis of immunological, viral quantification and genotype of acute and fulminant hepatitis A virus.

**Methods:**

Serum samples obtained from 1009 cases of suspected acute viral hepatitis was employed for different biochemical and serological examination. RNA was extracted from blood serum, reverse transcribed into cDNA and amplified using nested PCR for viral quantification, sequencing and genotyping. Immunological cell count from freshly collected whole blood was carried out by fluorescence activated cell sorter.

**Results:**

Fulminant hepatitis A was mostly detected with other hepatic viruses. CD8^+ ^T cells count increases in fulminant hepatitis to a significantly high level (P = 0.005) compared to normal healthy control. The immunological helper/suppressor (CD4^+^/CD8^+^) ratio of fulminant hepatitis was significantly lower compared to acute cases. The serologically positive patients were confirmed by RT-PCR and total of 72 (69.2%) were quantified and sequenced. The average quantitative viral load of fulminant cases was significantly higher (*P *< 0.05). There was similar genotypic distribution in both acute and fulminant category, with predominance of genotype IIIA (70%) compared to IA (30%).

**Conclusions:**

Immunological factors in combination with viral load defines the severity of the fulminant hepatitis A. Phylogenetic analysis of acute and fulminant hepatitis A confirmed genotypes IIIA as predominant against IA with no preference of disease severity.

## 1. Background

Hepatitis A virus (HAV) is one of the common causative agents for acute hepatitis worldwide, particularly in developing countries where 20-25% of clinical hepatitis is caused by HAV infection [1,2]. Hepatitis A is an acute infection with generalized symptoms accompanied by jaundice and it represents mainly a disease of the pediatric population [3,4]. In children, the infection with HAV is generally asymptomatic while exposure of non-immune adolescents and adults may results in severe clinical disease like fulminant hepatic failure (FHF) [2,5-7]. The pathogenetic mechanisms underlying hepatocellular injury in acute hepatitis are poorly understood [8]. There is general agreement that HAV infection does not evolve to chronic hepatitis in man and immune mechanisms have been suspected of playing a major role in eliminating virus infected liver cells [9,10].

**Figure 1:**
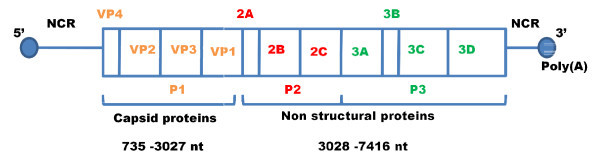
**Genomic structure of hepatitis A virus**. HAV genome is divided into a 5' non-coding region (5'NCR), a giant open reading frame, and a non-coding region (3'NCR). The coding region is subdivided into regions P1, P2 and P3. Adapted from: Totsuka and Moritsugu; Intervirology 1999; 42: 63.

HAV presents a spherical virion with 27 nm, with 7.5 kb linear, positive-sense RNA within the *Picornaviridae *family that demonstrates little antigenic variability [11,12]. HAV has been shown to possess a single conserved immunogenic neutralization site and isolates from different parts of the world belong to a single serotype [12,13]. It is composed of a 5' non-coding region (NCR), structural protein regions, non-structural protein regions and a 3' NCR [14,15]. HAV genome is of positive polarity, i.e., viral RNA can directly serve as messenger RNA [16]. The large open reading frame present in HAV genome can be divided into three (P1-P3) functional regions. The P1 region encodes capsid polypeptides VP1-VP3 and the putative VP4. The P2 and P3 regions encode non-structural proteins which are necessary for virus replication (Figure 1) [17]. The nucleotide sequence comparison based on the VP1/2A region of the genome has been used to define seven different genotypes [15]. The seven genotypes are differentiated into four human clusters (I-III and VII) and three simian strains (IV-VI) [15]. The seven genotypes recovered from human and non-human primates differ from each other at approximately 15 to 25% of base position in the VP1/2A region [15]. The VP1/2A region is best suited for genotyping due to its relative variability compared with the VP3 and 5' non-translated regions [15]. The nucleotide diversity within sub-genotypes of HAV (IA, IB, IIIA and IIIB) is less than 7.5% [15]. Most of the human strains cluster in genotype I, which has been further divided in sub-genotypes IA and IB. Sub-genotype IIIA had been reported to be the major HAV genotype in India [18-20]. However, recent report contradicts earlier findings that revealed sub-genotype IB in western India from samples of sewage treatment plant [19].

HAV is hyperendemic in India and most of the population is infected asymptomatically in early childhood with lifelong immunity [21]. However, recently due to altered epidemiology and decreasing endemicity the pattern of acute HAV infection is changing from asymptomatic childhood infection to an increased incidence of symptomatic disease in the adult population [21-23]. Although HAV is undergoing epidemiological changes in India, there are very few in-depth characterizations of all the causative factors related to HAV infection. Therefore, the aim of this study was to undertake an in-depth analysis of immunological, viral quantification and genotype of acute and fulminant hepatitis A virus.

## 2. Materials and methods

### 2.1 Patients and blood samples

All those patients attending the medical out patients department (OPD) of Lok Nayak Hospital, New Delhi, with the characteristic symptoms of jaundice, fever, general malaise, fatigue, nausea, vomiting, anorexia and right upper quadrant discomfort were enrolled in this study. This has been approved by the ethical committee of Maulana Azad Medical College, New Delhi, as directed by the Declaration of Helsinki in 1995. Ten milliliters of blood sample was taken by venipuncture after informed consent of the patients, while children and adolescents consent was obtained from their parents and guardians.

### 2.2 Diagnosis of acute and fulminant hepatitis A

The clinical onset of an acute viral hepatitis (AVH) A is defined as the beginning of early symptoms including fever, general malaise, fatigue, nausea, vomiting, anorexia and right upper quadrant discomfort. It is mainly characterized by the onset of jaundice and positive serological test for IgM anti-HAV. Fulminant hepatitis A is defined as clinical syndrome develops as a result of severe impairment of hepatic functions or massive necrosis of hepatocytes in the absence of preexisting liver disease. Fulminant hepatic failure (FHF) patients were diagnosed by the presence of encephalopathy within 4 weeks of onset of illness with a prothrombin time (PT) of less than 40% that of the standardized value [16,24].

### 2.3 Epidemiological survey

Each person enrolled in this study gave personal information including general data, such as gender, age, onset of illness, past history of hepatitis, alcoholism, and presence of major systemic disease. Those AVH patients who had positivity for any of IgM anti-HEV, hepatitis B surface antigen (HBsAg), IgM antibody to hepatitis B core antigen (IgM anti-HBc), and hepatitis C virus antibodies were excluded. Since fulminant hepatitis A is rare occurrence, we have reported all the FHF cases including cases with other hepatic viruses (60% in this study). Patients with a history of recent exposure to drugs and those who had a heavy alcohol intake were also excluded from the study. The travel history within 3 months before the onset of illness was carefully checked and recorded, as incubation period for HAV is 2-8 weeks.

### 2.4 Serological tests

A total of 1009 AVH and FHF patients were screened for IgM anti-HAV. In laboratory examination, serum albumin/globulin, alanine aminotransferase (ALT), aspartate aminotransferase (AST), alkaline phosphatase (ALP), total protein and total and direct bilirubin levels were measured. IgM anti-HAV was detected by enzyme immunoassay ELISA (HAVAB-MEIA, Abbott Laboratories, North Chicago, IL) according to the manufacturer's instructions. To determine other acute viral hepatitis, sera were also tested for IgM anti-HEV (Qiagen, Hilden, Germany), hepatitis B surface antigen (Qiagen, Hilden, Germany), IgM anti-HBc (Abbot Laboratories, North Chicago, IL), anti-HCV (Qiagen, Hilden, Germany) using Kits.

### 2.5 FACS analysis of T-lymphocyte profile

One milliliter of whole blood was collected from patients into a vial containing EDTA and was employed for CD4^+ ^(T helper) and CD8^+ ^(T suppressor) cell counts within 24 hours of collection using fluorescence activated cell sorter (FACS) (Becton Dickenson Electronics Laboratory, Mountain View, California). This system quantifies CD4^+^, CD8^+ ^and CD3^+ ^T lymphocytes as absolute numbers of lymphocytes per μl (mm^3^) of blood, and the CD4^+^/CD8^+ ^T lymphocyte ratio. Samples from healthy controls were also run for cell counts using the manufacturer's protocol and reagents.

### 2.6 HAV RNA detection

RNA was extracted from each test sample, using the QIAamp viral RNA extraction kit (Qiagen, Germany) and reverse transcribed into cDNA using published primer [25]. The cDNA was then amplified in a nested PCR targeting VP1/2A region as shown in figure 1[26]. Nested PCR was carried out using primers BR-5b (5'-TTG TCTGTC ACA GAA CAA TCAG-3') as the outer, sense primer, BR-9b as the outer, anti-sense primer, RJ-3c (5'-TCC CAG AGC TCC ATT GAA-3') as the inner, sense primer, and Br-6b (5'-AGG AGG TGG AAG CAC TTC ATT TGA-3') as the inner, anti-sense primer [25]. Both amplification reactions were performed in a 9600 thermo-cycler (Perkin-Elmer Cetus, Norwalk, CT) set to run for 2 min at 95°C (denaturation), 1 min at 55°C (primer annealing) and 1 min at 72°C (extension) for 35 cycles, with a final extension step at 72°C for 10 min. After electrophoresis in 2% agarose (Research Organics, Cleveland, OH) and staining with ethidium bromide, a ultra-violet transilluminator (Gel Doc 1000, Bio-Hercules, CA) was used to check for the expected, 234 bp band.

### 2.7 Direct sequencing

The target PCR products within the agarose gel were purified for sequencing using the perfect prep Gel Cleanup Kit (Eppendorf, Westbuty, NY), according to the manufacturer's specifications, and subjected to 2% agarose gel electrophoresis in order to ascertain their purity. Between 10 and 30 ng/μl (3-6 μl) of each DNA sample was subjected to cycle sequencing using 8 μl of dye terminator from a DNA sequencing kit (Big Dye Terminator V.3.0 Cycle Sequencing Ready Reaction, Foster City, CA) and 3.2 pmole of specific primer (in a final reaction volume of 20 μl) in a thermocycler (9600 Perkin-Elmer Cetus, Norwalk, CT). This round of amplification was performed according to the manufacturer's specifications, using primer BR-5b to amplify the particular DNA strand of interest for further sequencing. The extension products were subsequently purified from excess unincorporated dye terminators by ethanol precipitation, according to the manufacturer's specifications (ABI Sequencing kit, ABI, Foster City, CA), and subjected to sequence analysis by the ABI Prism 310 Genetic Analyzer (ABI, Foster City, CA).

### 2.8 Primers, probe and standard for real-time amplification

Viral RNA was amplified using primers derived from the most constant region, the 5' non-coding region (5'NCR) as shown in figure 1. The primers used were, forward primer HAV-1 (5'-TTTCCGGAGCCCCTCTTG-3'), as wild type (M14707) reverse primers HAV-2 (5'-AAA GGGAAATTTAGCCTATAG CC-3') and HAV-3 (5'-AAAGGGAAAATTTAGCCTATA GCC-3'), and HAV-Probe (5'-FAM-ACTTGATACCTCACCGCCGTTTGCCT-TAMRA-3') and RNA standard representing the 5'NCR region was constructed according to Costa-Mattioli *et al*. [27]. RT-PCR was carried out with a HAV quantification kit (Roche Diagnostics GmbH, Germany) according to the manufacturer's instructions. The total volume of the reaction mixture was 25 μl (15 μl of master mix with 10 μl of the RNA template) in 0.2 ml tubes. The capillaries were sealed, centrifuged, and transferred to the Rotor Gene 3000 real-time PCR machine (Corbett Research, Sydney, Australia). Reverse-transcription was done for 15 min at 50°C followed by 5 min denaturation at 95°C. The corresponding cDNA's were amplified by PCR (20s at 95°C, 30 s at 50°C acquiring FAM, and 20 s at 72°C) over 45 cycles, and an 87 bp fragment was obtained. The CT values from the clinical samples were plotted on the standard curve, and the number of copies was calculated automatically.

### 2.9 Phylogenetic analysis and genotype determination

The HAV genome shows a high degree of stability, but the VP1/2A junction region is recognized as one of the most variable regions. The location of VP1/2A junction region in HAV genome is shown in Figure 1, and chosen for genotyping and phylogenetic analysis. Website http://www.phylogeny.fr was used for reconstructing and analyzing phylogenetic relationships between different HAV Indian isolates. The HAV Indian isolates were amplified and sequenced from clinical samples and compared with the corresponding GeneBank reference sequences for genotypes I-III, IV, VI, & VII are provided in additional file 1. The GeneBank sequence accession numbers of studied hepatitis A virus sequences which constitute phylogenetic tree are incorporated in figure legends 4 and 5 and published sequences in additional file 2.

### 2.10 Statistical analysis

Chi-squared analysis, Fisher's exact test, Student's *t*-test, Mann-Whitney *U*-test, Wilcoxin two-sample test, ANOVA and stepwise backward Cox regression procedure were used in this study. A *P *value of <0.05 was considered significant.

## 3. Results

Total of 1009 acute and fulminant viral hepatitis cases were screened during the period of 5 years, 556 were males and 453 females with sex ratio 1.2:1. The mean age and standard deviation of the patients were 28.2 ± 21.8 years.

### 3.1a Serological screening of acute and fulminant hepatitis A

Out of one thousand and nine hundred acute cases, 104 (10.3%) were positive for IgM anti-HAV by commercially available ELISA kit. Out of 104 HAV-IgM positive cases, 10 (0.96%) developed into fulminant hepatitis A. Other viral etiologies were detected in 60% of HAV fulminant cases as shown in Table 1.

**Table 1:** Acute and fulminant HAV patients infected with other etiological agents

Etiological agents	**AVH**^**a**^ (n = 94)	**FHF**^**b**^ (n = 10)
HBsAg ^c^+ IgM anti-HBc ^d^	03	02
Anti-HCV ^e^	--	01
HEV-IgM ^f^	05	--
HEV-IgM ^f ^+ HBsAg ^c^	--	01
HEV-IgM ^f ^+ Anti-HCV ^d^	--	02

^**a **^**AVH **= Acute viral hepatitis; ^b ^**FHF **= Fulminant hepatic failure; ^**c **^HBsAg = hepatitis B surface antigen; ^**d **^IgM anti-HBc = Hepatitis B core antibody; ^**e **^Anti-HCV = Hepatitis C virus antibody; ^**f **^HEV-IgM = Hepatitis E virus antibody

### 3.1b Sensorium/hepatic encephalopathy of fulminant hepatitis A

Fifty percent of the fulminant hepatitis A patients fall under clinical grade I hepatic encephalopathy. These patients were restless and had almost uncomplicated course and recovered completely. One patient in grade II hepatic encephalopathy was restless and confused but recovered finally. Four patients died of hepatic encephalopathy, three due to grade IV hepatic encephalopathy. These patients were in coma and cannot be aroused. While patient of hepatic encephalopathy grade III developed drowsy and confused state and died due to complications. The duration of hepatic encephalopathy among survivors and non-survivors were similar (*P *> 0.03). Encephalopathy developed within 1-4 weeks of onset of symptoms, and mean duration between onset of symptoms and encephalopathy was 6.3 ± 0.3 days.

### 3.1c Biochemical profile of acute and fulminant hepatitis A

Mean haemoglobin (g/dl) was significantly lesser in acute and fulminant hepatitis A compared to healthy control, while difference between acute and fulminant hepatitis A cases was statistically significant (*P *< 0.035). Similarly, prothrombin time was higher among fulminant cases compared to acute viral hepatitis A (*P *< 0.04) as shown in additional file 3. The liver function profiles of acute and fulminant hepatitis A at different days of follow up as shown in additional file 4. Liver function profiles (ALT, AST and ALP) of fulminant hepatitis A at different days of follow up were significantly higher compared to acute viral hepatitis A. In acute cases the above values decreased from initial to final days but not FHF. Total bilirubin was similar (P = 0.55) at initial day, but at 1^st ^week of the onset of jaundice total bilirubin was quite higher (P = 0.002) in the fulminant category compared to the acute cases. The rest of the liver function parameters (TB, DB/IB, ALP, TP, and Alb) were not statistically significant.

### 3.4 Epidemiological profile of acute and fulminant hepatitis A

The epidemiological characterization of acute and fulminant hepatitis was similar with no significance. Twenty one patients (33.8%) in the acute category had history of being exposed to unhealthy surroundings with poor sanitation facilities compared to 3 (30%) in fulminant hepatitis A as shown in Table 2. Travel to high endemic area was also found to be a major cause that leads to spread of hepatitis A virus. Eight (13%) acute cases and one (10%) fulminant hepatitis A patient had travel history (P = 0.98).

**Table 2:** Epidemiological characteristics of acute and fulminant hepatitis A

Characteristics	**AVH**^**a**^ (n = 94)	**FHF**^**b**^ (n = 10)
Travel history	13 (13.8%)	1 (10%)
Unhygienic condition	32 (34.0%)	3 (30%)

^**a **^AVH = Acute viral hepatitis; ^**b **^FHF = Fulminant hepatic failure

**Travel history**; AVH vs FHF: P = 0.98 **Unhygienic condition**; AVH vs FHF: P = 0.87. Mann-Whitney *U*-test was applied to find the statistical difference between the above groups. *P *value <0.05 is statistically significant, so the *P *value is not significant

### 3.5 Immunological profiles of acute and fulminant hepatitis A

**Figure 2:**
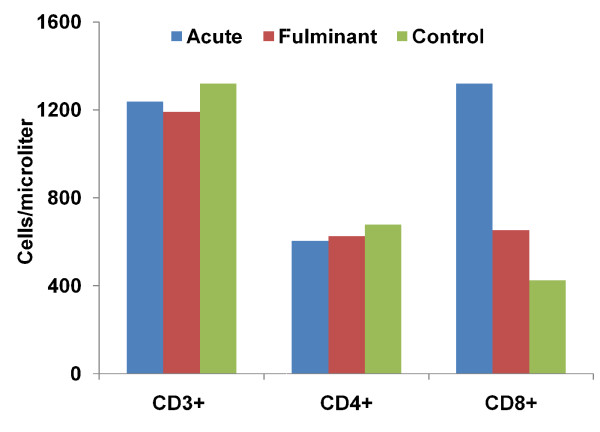
**FACS analysis of T lymphocytes showing lymphocytes counts acute (AVH), fulminant (FHF) and normal healthy control (C)**. Mann-Whitney *U*-test was applied to find the statistical difference between the above groups. **CD3**^**+**^; AVH vs FHF: **P **= 0.864, AVH vs C: **P **= 0.297, FHF vs C: **P **= 1.000 **CD4**^**+**^; AVH vs FHF: **P **= 0.434, AVH vs C: **P **= 0.086, FHF vs C: **P **= 0.135, **CD8**^**+**^; AVH vs FHF: **P **= 0.244, AVH vs C: **P **= 0.074, FHF vs C: **P **= 0.000.

**Figure 3:**
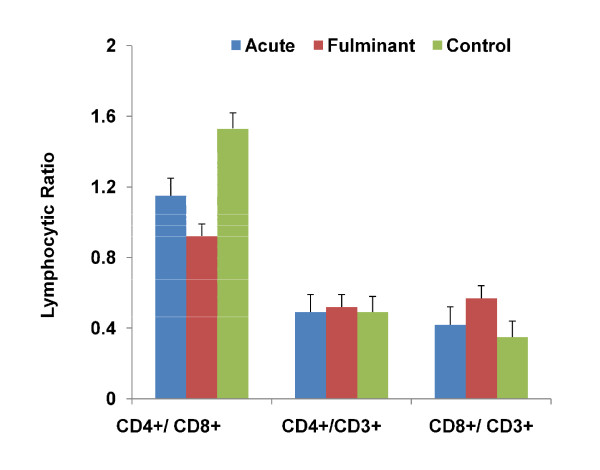
**FACS analysis of T lymphocytes showing lymphocytic ratio (CD4**^**+**^**/CD8**^**+**^**) of acute (AVH), fulminant (FHF) and normal healthy control (C)**. Mann-Whitney *U*-test was applied to find the statistical difference between the above groups. **CD4**^**+**^**/CD8**^**+**^; AVH vs FHF: **P **= 0.038, AVH vs C: **P **= 0.063, FHF vs C: **P **= 0.000 **CD4**^**+**^**/CD3**^**+**^; AVH vs FHF: **P **= 0.366, AVH vs C: **P **= .029; FHF vs C: **P **= 0.818 **CD8**^**+**^**/CD3**^**+**^; AVH vs FHF: **P **= 0.112, AVH vs C: **P **= 0.08, FHF vs C: **P **= 0.018.

The lymphocyte sub-population (i.e. CD4^+ ^(helper)/CD8^+ ^(suppressor) ratio of acute and fulminant hepatitis A was compared with normal healthy control. CD8^+ ^T cells count increases in fulminant and acute hepatitis cases compared to normal healthy control, but this increase was significant only in the first case (P = 0.005 and 0.074). In acute and fulminant hepatitis cases, CD4^+ ^T cells concentration (cells/microliters) maintained a low level compared to normal healthy control as shown in Figure 2. In fulminant hepatitis cases helper/suppressor T-lymphocyte ratio decreased to a significant quantity compared to the ratio observed among normal control as shown in Figure 3. CD8^+ ^(cytotoxic T lymphocytes) concentration (cells/microliters) increases to target the infected (hepatocytes), while CD4^+ ^T lymphocytes decline, resulting in the helper suppressor ratio and hence immunity.

### 3.6 Molecular analysis of serologically positive samples

All IgM anti-HAV positive samples (104) were analyzed for HAV RNA presence to correlate the time of presentation of the disease with HAV-RNA positivity. Fifty eight (56%) of AVH cases turned positive for RT-PCR, reported to OPD at 0-7 days of onset of jaundice. This includes fulminant hepatic failure following the acute illness. The positivity by RT-PCR in acute and fulminant was 75% and 100% respectively as shown in Table 3. HAV RNA detection was higher in fulminant hepatitis cases compared to that observed among acute cases. Thirty two patients positive for hepatitis A by ELISA turned to OPD in 2^nd ^week of the onset of jaundice. Again the positivity of fulminant case was 100%, while in acute case the positivity declined to 62%. The RNA positivity of patients who turned to the OPD in 1^st ^and 2^nd ^months of the onset of jaundice again declined. There was significant difference (*P *> 0.05) between final positivity of acute and fulminant hepatitis. All the positive acute and fulminant cases were directly sequenced to determine the genotype(s).

**Table 3:** Correlation between HAV RNA positivity with respect to duration of jaundice

Duration of Jaundice	**HAV RNA AVH**^**a**^ (n = 62)	**HAV RNA FHF**^**b**^ (n = 10)
0-7 days	38/51(74.5%)	07/07 (100%)
7-14 days	18/29 (62.0%)	03/03 (100%)
1-2 months	05/11 (45.4%)	--
5 months	01/03 (33.3%)	--

^**a **^AVH = Acute viral hepatitis; ^**b **^FHF = Fulminant hepatic failure

There was significant difference (*p *< 0.05) between final positivity of acute and fulminant hepatitis A.

### 3.6a Phylogenetic analysis and genotype distribution of hepatitis A virus

**Figure 4:**
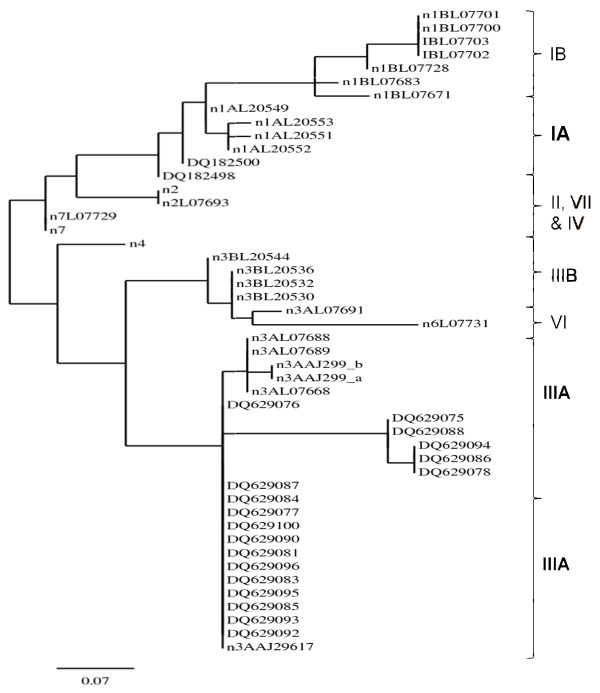
**A neighbor-joining phylogenetic tree of genetic relatedness among isolates of hepatitis A virus based on the sequencing of the VP1/2A region. Isolates DQ182500, DQ182498, DQ629076, DQ629075, DQ629088, DQ629094, DQ629086, DQ629078, DQ629087, DQ629084, DQ629077, DQ629100, DQ629090, DQ629081, DQ629096, DQ629083, DQ629095, DQ629085, DQ629093 and DQ629092 were collected during this study, in PCR Hepatitis Laboratory, MAM College and associated LNJ Hospital, New Delhi, India**. The phylogenetic analysis revealed that the Indian isolates belong to genotypes IA and predominant III A.

**Figure 5:**
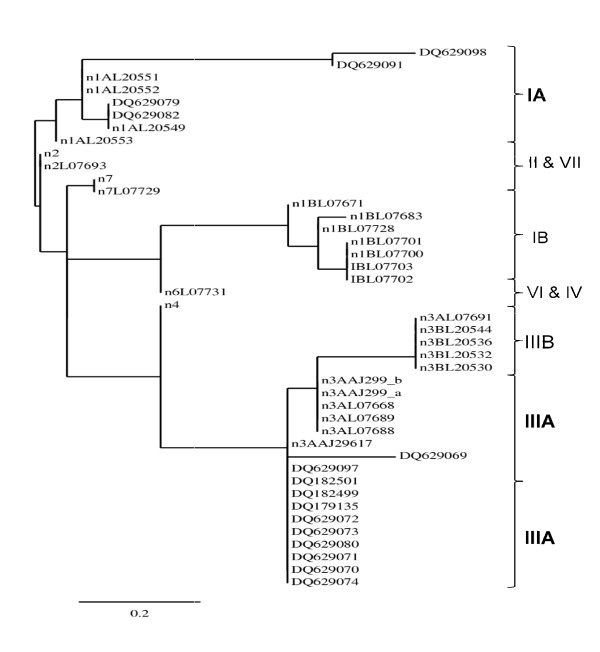
**A neighbor-joining phylogenetic tree of genetic relatedness among isolates of hepatitis A virus based on the sequencing of the VP1/2A region. Isolates DQ629098, DQ629091, DQ629079, DQ629082, DQ629069, DQ629097, DQ182501, DQ182499, DQ179135, DQ629072, DQ629073, DQ629080, DQ629071, DQ629070 and DQ629074 were collected during this study, in PCR Hepatitis Laboratory, MAM College and associated LNJ Hospital, New Delhi, India**. The phylogenetic analysis revealed that the Indian isolates belong to genotypes IA and predominant III A.

Accommodating large number of sequenced samples (reference and studied sequences) in one phylogenetic tree lacks clarity, therefore for most accurate presentation 2 phylogenetic trees have been constructed as shown in Figure 4 and 5. The genotype of Indian HAV isolates was derived by neighbor-joining phylogenetic tree of hepatitis A virus sequences based on the VP1/2A region. According to phylogenetic analysis, acute cases were divided into genotype IA and IIIA. There was predominance of genotype IIIA with 74% of the total cases sequenced. In fulminant hepatitis A cases, genotype IIIA again was also prevalent (70%) as shown in Table 4. Between the groups the genotype was similarly distributed but within the two groups the prevalence was quite significant.

**Table 4:** Comparison of genotype(s) between acute and fulminant hepatitis A patients

**AVH**^**a **^**(n = 62)**		**FHF**^**b **^**(n = 10)**	
Genotype (s)		Genotype (s)	
IA	IIIA	IA	IIIA
16 (26%)	46 (74%)	03 (30%)	07(70%)

^**a **^AVH = Acute viral hepatitis; ^**b **^FHF = Fulminant hepatic failure

Fisher's exact test was applied to find the statistical difference between the acute and fulminant groups. *P *value calculated between the two groups was 0.52, the value was statistically insignificant.

### 3.7 Viral load determination among acute and fulminant hepatitis A

**Figure 6:**
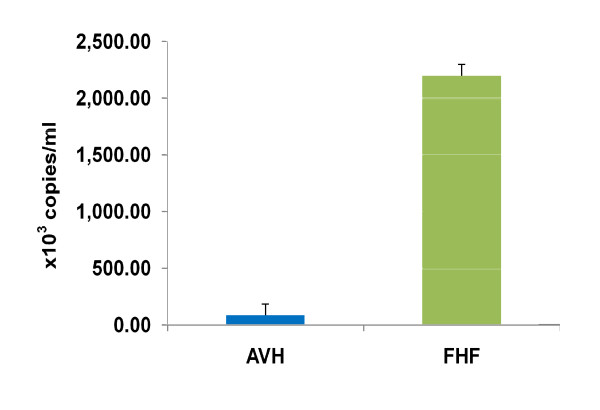
**Viral load determination of acute and fulminant hepatitis A by real time PCR**. Mann-Whitney *U*-test was applied to find the statistical difference between the above groups. Quantitative viral load; AVH vs FHF: P = 0.0000; highly significant.

All the patients (acute and fulminant hepatitis A) were quantified for viral load by real-time polymerase chain reaction. As shown in Figure 6, HAV viral load among fulminant cases was much higher compared to acute viral hepatitis A. According to the non parametric Mann-Whitney *U*-test, the calculated viral load concentration of the fulminant patients was significantly quite higher compared to the acute viral hepatitis A patients.

## 4. Discussion

Human hepatitis A, a widespread infectious disease that is hyperendemic in vast areas of the world, results from the infection of the liver [3,28]. Humans are thought to be its principal host and represent a disease of pediatric population [28]. Importantly, one third of the patients in present study belong to age group 11-30 years which is quite different from the earlier findings that pediatric populations are more prominent [21]. HAV distribution pattern was in corroboration with earlier findings which showed male predominance and susceptibility to HAV infection compared to female population [3,28]. The present study showed that one third of hepatitis A patients had insufficient sanitation or poor hygienic conditions with overcrowded population. As reported earlier, low standards of sanitation promote transmission of the virus, therefore, to avoid any outbreak of this infection in new entrants there is urgent need for proper sanitation [29,30]. Traveler's account for 10-13% of the acute and fulminant hepatitis A in this study, similar to the earlier reports which estimate an annual infection rate of 3.5-7.2 per 100 individuals [18,31].

40% of death (non-survivors) and complications were related among hepatitis A cases presenting histology of III and IV, what was also demonstrated in other studies [6,7]. Most of the non-survivors were infected with other viral etiologies. The survivors of fulminant hepatitis A belong to Grade I and II with no infection with other viral hepatitis. Prothrombin time (PT) of fulminant hepatitis A cases were significantly prolonged compared to normal controls (P = 0.035). The reason why fulminant hepatitis A occurs in some patients and not in other remains unclear. HAV infection rarely has a fulminant course and is seldom fatal, with an estimated fatality rate of 0.14 to 2.0 percent [8], which corroborates the findings of the present study. Diagnosis of hepatitis A were usually made by LFTs and followed at different interval of time [31,32]. ALT, AST and ALP levels of fulminant viral hepatitis cases at different days of follow up was significantly higher compared to acute hepatitis A. This was similar to earlier findings that aminotransferase levels are sensitive markers of liver damage, and usually reach peak levels about the time the patients seek medical attention, although the degree of elevation does not appear to correlate the outcome [32-34].

The clearance of viral infection and the disease manifestations are associated with cellular immune response [34]. The relative percentage of CD4^+ ^T lymphocytes decline and CD8^+ ^T lymphocytes increases in the fulminant HAV cases results in decline of the helper/suppressor ratio. This decrease of CD4^+^/CD8^+ ^T lymphocyte ratio was highly significant compared to acute cases (P = 0.038). This shows that low CD4^+ ^T lymphocyte counts are associated with a variety of conditions in viral infections [35]. These findings were in corroboration with study carried out on patients with cervical intraepithelial neoplasia and invasive cancer revealed a decrease in CD4 cells with a relative increase in CD8 cell count, leading to a considerable reduction in the CD4/CD8 cell ratio [36]. Therefore the present study speculate that fulminant HAV infection may be triggered by diminished cellular immunity in susceptible patients and increase liver damage and hence fatality and severity.

The detection rate and mean duration of HAV viremia was 69.3% and 15 days (range 1-2 months) respectively [1]. This proves earlier findings that HAV RNA could be detected on an average 18 days following the onset of clinical symptoms [32,34]. The average viral load of fulminant hepatitis was significantly higher compared to the acute hepatitis A cases in the present study. Although HAV is not directly cytopathic, viral factor might be one reason involved in determining the severity of the disease [37]. Seven HAV genotypes have been defined based on the sequence of the VP1/2A junction region of a global collection of viruses [15]. HAV isolates reported from India were found to be of genotype IIIA [15]. However recent study from western India demonstrated presence of genotype IB [19]. Vaidya et al., study had limitation due to the collection of HAV isolates from sewage treatment plant and not directly from the patients [19]. The current study detected HAV RNA by using RT-PCR in the sera of patients instead of feces. The acute hepatitis A genotype (s) distribution was found to be IA (26%) and IIIA (74%). Similarly, fulminant hepatic genotype distribution was IA (30%) and IIIA (70%) with no significant difference between these groups (P = 0.52). Thus, it appears that the prevalence of HAV genotypes in Northern India is was different from western India, indicating there could be geographical variations in the prevalence of HAV genotypes [15,18,19].

## 5. Conclusions

In summary, results suggest that hepatitis A is a major public health problem in India. Further, most hepatitis A cases reported was from poor hygienic surroundings, which emphasizes the need for improving the public health measures to prevent epidemics of hepatitis A. There was relative percentage of CD4^+ ^T lymphocytes decline and CD8^+ ^T lymphocytes increase in the fulminant HAV. This results in decline in helper/suppressor ratio that might lead to development of a weak antiviral immune response or diminished cellular immunity to the viral antigens. Significant increase of viral copies in fulminant patients defines its role in determining the severity of the disease. Phylogenetic analysis of acute and fulminant hepatitis A confirmed genotypes IIIA as predominant against IA with no preference of disease severity. Finally, both viral and host factors should be considered and examined when discussing the mechanisms responsible for the severity of type A hepatitis.

## 6. Competing interests

The authors declare that they have no competing interests.

## 7. Authors' contributions

ZH, PK participated in study design. ZH participated in sample collection, experimentation. ZH, SAH, and PK participated in data analysis. FNA edited the manuscript. All authors read and approve the final manuscript.

## Supplementary Material

Additional file 1 **GeneBank reference sequences**. The sequenced HAV north Indian isolates were compared with different reference sequences representing genotypes: I-III, IV, VI, & VII. Click here for file

Additional file 2 **Hussain *et al*., published sequences with GeneBank accession number**. Hussain *et al*., published sequences which were categorized into genotype IA and IIIA. These north Indian isolates of hepatitis A virus were characterized based on the sequencing of the VP1/2A region. Click here for file

Additional file 3 **Haemoglobin and prothrombin time of acute and fulminant hepatitis A**. The mean haemoglobin and prothrombin time of acute and fulminant hepatitis A patients were compared with the normal healthy control. Click here for file

Additional file 4 **The follow up of liver function profile of acute and fulminant hepatitis A**. Liver function profile of acute and fulminant hepatitis A patients were followed at different days and weeks. Click here for file
