# Supplementary material for: Immunological and molecular epidemiological characteristics of acute and fulminant viral hepatitis A
Source: Virol J. 2011 May 23;8:254. doi: 10.1186/1743-422X-8-254 (PMC3117845; doi:10.1186/1743-422X-8-254)
Supplement: Additional file 1 — GeneBank reference sequences. The sequenced HAV north Indian isolates were compared with different reference sequences representing genotypes: I-III, IV, VI, & VII. [file 1743-422X-8-254-S1.DOCX]

**Additional file 1**

Title: GeneBank reference sequences

Description: The sequenced HAV north Indian isolates were compared with different reference sequences representing genotypes: I-III, IV, VI, & VII.

L07683, L07671, L07703, L07702, L07701, L07728, L07700 (IB), L20553, L20552, L20551, and L20549 (IA), L07693 (II), L07729 (VII), L07691, L20544, L20536, L20532, L20530 (IIIB), L07731 (VI), AJ299466, AJ299464, AJ296172.1, L07688, L07689 (IIIA)
