# Supplementary material for: Immunological and molecular epidemiological characteristics of acute and fulminant viral hepatitis A
Source: Virol J. 2011 May 23;8:254. doi: 10.1186/1743-422X-8-254 (PMC3117845; doi:10.1186/1743-422X-8-254)
Supplement: Additional file 2 — Hussain et al., published sequences with GeneBank accession number. Hussain et al., published sequences which were categorized into genotype IA and IIIA. These north Indian isolates of hepatitis A virus were characterized based on the sequencing of the VP1/2A region. [file 1743-422X-8-254-S2.DOCX]

**Additional file 2**

Title: Hussain *et al.*, published sequences with GeneBank accession number

Description: Hussain *et al.*, published sequences were categorized into genotype IA and IIIA. These north Indian isolates of hepatitis A virus were characterized based on the sequencing of the VP1/2A region.

AY251007, AY251008, AY251009, AY251010, AY259831, AY599877, AY599878, AY599879, AY599880, AY599881, AY599882, AY618259, AY618260, AY618261, AY618262, AY618263, AY804171, AY804175, AY804174, AY804172, AY804173, AY804177, AY804178, AY804179, AY804176, AY804180, AY804181, DQ179131, DQ179132, DQ179133, DQ179134, DQ179135, DQ179136, DQ179137, DQ182495, DQ182496, DQ182497
