# Supplementary material for: Immunological and molecular epidemiological characteristics of acute and fulminant viral hepatitis A
Source: Virol J. 2011 May 23;8:254. doi: 10.1186/1743-422X-8-254 (PMC3117845; doi:10.1186/1743-422X-8-254)
Supplement: Additional file 3 — Haemoglobin and prothrombin time of acute and fulminant hepatitis A. The mean haemoglobin and prothrombin time of acute and fulminant hepatitis A patients were compared with the normal healthy control. [file 1743-422X-8-254-S3.DOCX]

**Additional file 3**

Title: Haemoglobin and prothrombin time of acute and fulminant hepatitis A

Description: The mean haemoglobin and prothrombin time of acute and fulminant hepatitis

A patients were compared with the normal healthy control.

Haemoglobin and prothrombin time of acute and fulminant hepatitis A

| Parameters | AVH | FHF | Normal Control |
| --- | --- | --- | --- |
| Haemoglobin (g/dL) | 11.4±2.3 | 9.7±2.0 | 13.5± 0.3 |
| Prothrombin time (s) | 14.3±5.2 | 17.5±2.0 | 13.0±0.5 |

**Haemoglobin:** AVH vs FHF P=0.035; **Prothrombin time:** AVH vs FHF P=0.04

*P* value <0.05 is statistically significant and values were expressed in mean ± SD
