# Supplementary material for: Immunological and molecular epidemiological characteristics of acute and fulminant viral hepatitis A
Source: Virol J. 2011 May 23;8:254. doi: 10.1186/1743-422X-8-254 (PMC3117845; doi:10.1186/1743-422X-8-254)
Supplement: Additional file 4 — The follow up of liver function profile of acute and fulminant hepatitis A. Liver function profile of acute and fulminant hepatitis A patients were followed at different days and weeks. [file 1743-422X-8-254-S4.DOCX]

**Additional file 4**

Title: The follow up of liver function profile of acute and fulminant hepatitis A

Description: Liver function profile of acute and fulminant hepatitis A patients were followed at different days and weeks.

## LFT profiles of acute and fulminant A patients at different days/weeks of follow up

| Days/Weeks  Parameters | 0 day | 1^st^ week | 2^nd^ week |
| --- | --- | --- | --- |
| ^1^AST AVH^a^    FHF^b^ | 886.0 ± 1003.3  1748.7 ± 1280.5 | 774.7 ± 903.1  1018.4 ± 1240.8 | 364.7 ± 419.5  1422.6 ± 2113.0 |
| ^2^ALT AVH    FHF | 1035 ± 1261.9  2592.4 ± 2117.0 | 750.3 ± 948.4  1570.8 ± 1233.2 | 452.8±783.9  1592.3±1438.6 |
| ^3^TB AVHFHF | 7.56 ± 5.37  8.78 ± 5.17 | 3.70 ± 3.19  8.6 ± 6.6 | 3.38 ± 2.66  5.12 ± 3.74 |
| ^4^DB AVH/FHF    IB AVH/FHF | 3.1±1.3/3.9±3.3  2.3±1.4/3.2±2.5 | 2.2±1.2/3.5±3.4  1.8 ±0.7/2.3±1.8 | 1.7±1.4/2.02±1.6  1.2 ±0.8/1.9±1.3 |
| ^5^ALP AVH    FHF | 303.01± 258.15  480.6 ±396.6 | 234.0 ± 203.1  342.3±278.2 | 167.3±134.3  203.8±145.0 |
| ^6^TP AVH    FHF | 6.62 ± 0.67  6.66 ± 0.88 | 6.55 ± 0.55  6.41 ± 0.49 | 6.34 ± 0.57  6.23±0.67 |
| ^7^Alb AVH    FHF | 3.76 ± 0.86  3.45 ± 0.86 | 3.78 ± 0.85  3.46 ± 0.86 | 3.82±0.78  3.29±0.56 |

^a^ AVH= Acute viral hepatitis ; ^b^ FHF= Fulminant hepatic failure

**^1^AST**; Serum glutathione aminotransferase: AVH vs FHF P=0.042 (0^th^ day), P=0.052 (1^st^ week), P=0.046 (2^nd^ week) **^2^ALT**; Serum glutathione aspartate aminotransferase: AVH vs FHF P=0.018 (0^th^ day), P=0.028 (1^st^ week), P=0.45 (2^nd^ week) **^5^ALP;** Alkaline phosphatase: AVH vs FHF P=0.037 (0^th^ day), P=0.02 (1^st^ day) **^3^TB**; Total Bilirubin: P=0.002 (1^st^ week). Mann-Whitney *U*-test was applied and the difference was found to be significant, rest of the group **^3^TB**: Total Bilirubin; **^4^DB/IB**: Direct Bilirubin/ Indirect Bilirubin; **^6^TP**: Total protein; **^7^Alb**: Albumin was not significant (*P*>0.05). Mann-Whitney *U*-test was applied to find the statistical difference between the above groups.
